# Supplementary material for: Single-cell analysis identifies dynamic gene expression networks that govern B cell development and transformation
Source: Nat Commun. 2021 Nov 25;12:6843. doi: 10.1038/s41467-021-27232-5 (PMC8617197; doi:10.1038/s41467-021-27232-5)
Supplement: Supplementary file 1 — Supplementary Information [file 41467_2021_27232_MOESM1_ESM.pdf]

Single-cell analysis identifies dynamic gene expression networks that govern B cell development and transformation

Supplementary Information File

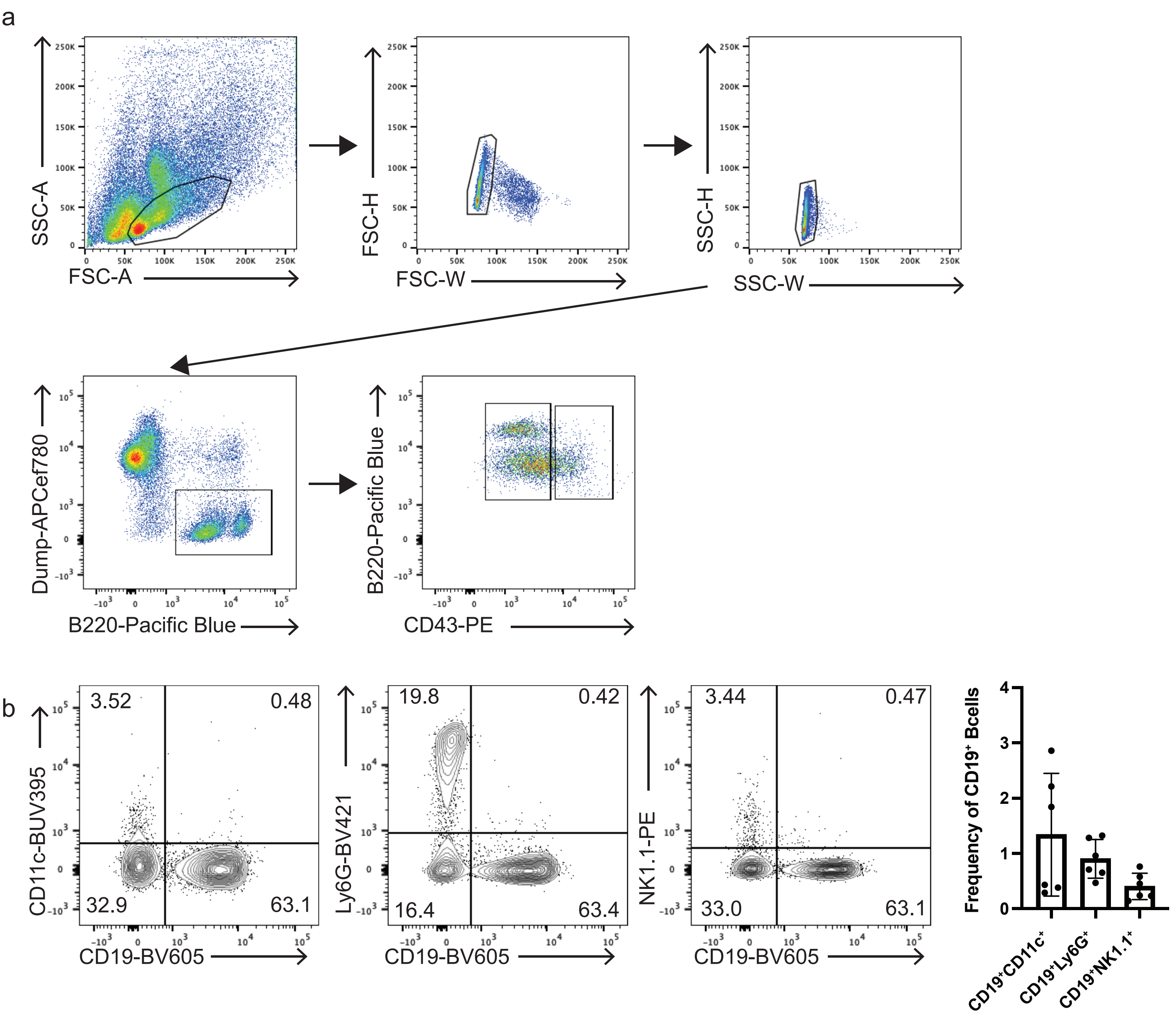

Supplementary Figure 1. Flow cytometry gating scheme and excluded B cells. a. Single cell lymphocytes are gated using FSC-A, SSC-A, FSC-H, FSC-W, SSC-H and SSC-W. B220$^+$Dump$^-$ (Dead, Ter119, NK1.1, Gr1, CD4, CD8, CD11c) were gated on to separate the early progenitor B cells (CD43$^+$) from the later progenitor B cells (CD43$^-$) for 1:1 sorting. **b.** Flow cytometry analysis of CD19$^+$ B cells that may have been excluded from downstream analysis due to sorting scheme. Expression of CD19 and CD11c, Ly6G, or NK1.1 in the bone marrow (left). Percentage of CD19+ B cells that expressed CD11c, Ly6G or NK1.1 (right). Data are presented as mean values ± S.D and *n* = 6 biological independent mice from three independent experiments.

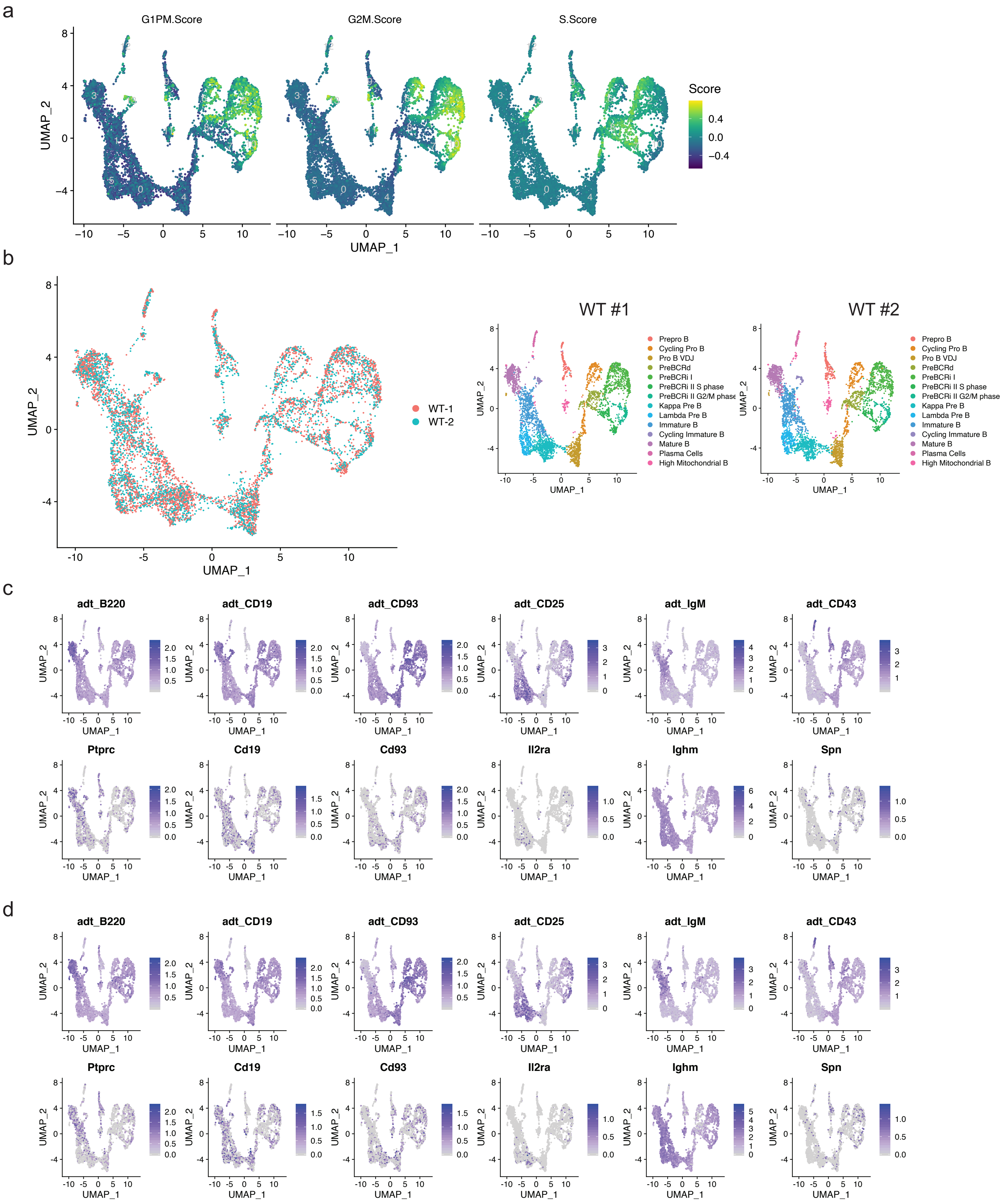

Supplementary Figure 2. Cell cycle scoring and wild type biological replicate representation. a. UMAP of the aver- age gene expression scores of different cell cycle stages (G1PM; G1 post-mitotic, or G2/M and S phase). **b.** UMAP plots showing cells colored based on origin of wildtype sample (left) or colored by cluster assignment and separated according to each wildtype sample (right). **c.** ADT/CITE-Seq antibody expression in wildtype sample #1 cells. **d.** ADT/CITE-Seq antibody expression in wildtype sample #2 cells. Color scale in c, d represents natural log transformed SCTrans- form corrected counts.

a

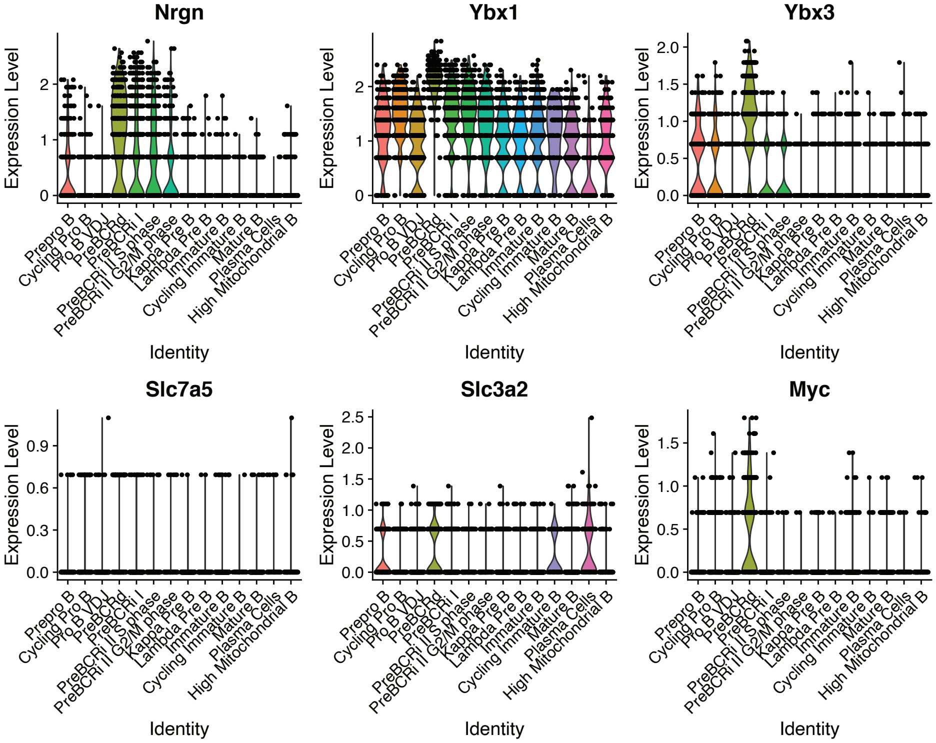

b

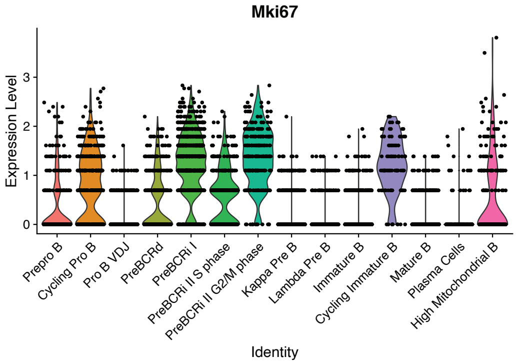

Supplementary Figure 3. Pre-B cell expansion gene expression. a. Violin Plot gene expression of Pre-BCR-de- pendent stage genes (Nrgn, Ybx1, Ybx3, Slc7a5, Slc3a2, Myc). **b.** Mki67 expression Violin Plot for all B cell development stages. Expression levels in a,b represent natural log transformed SCTransform corrected counts.

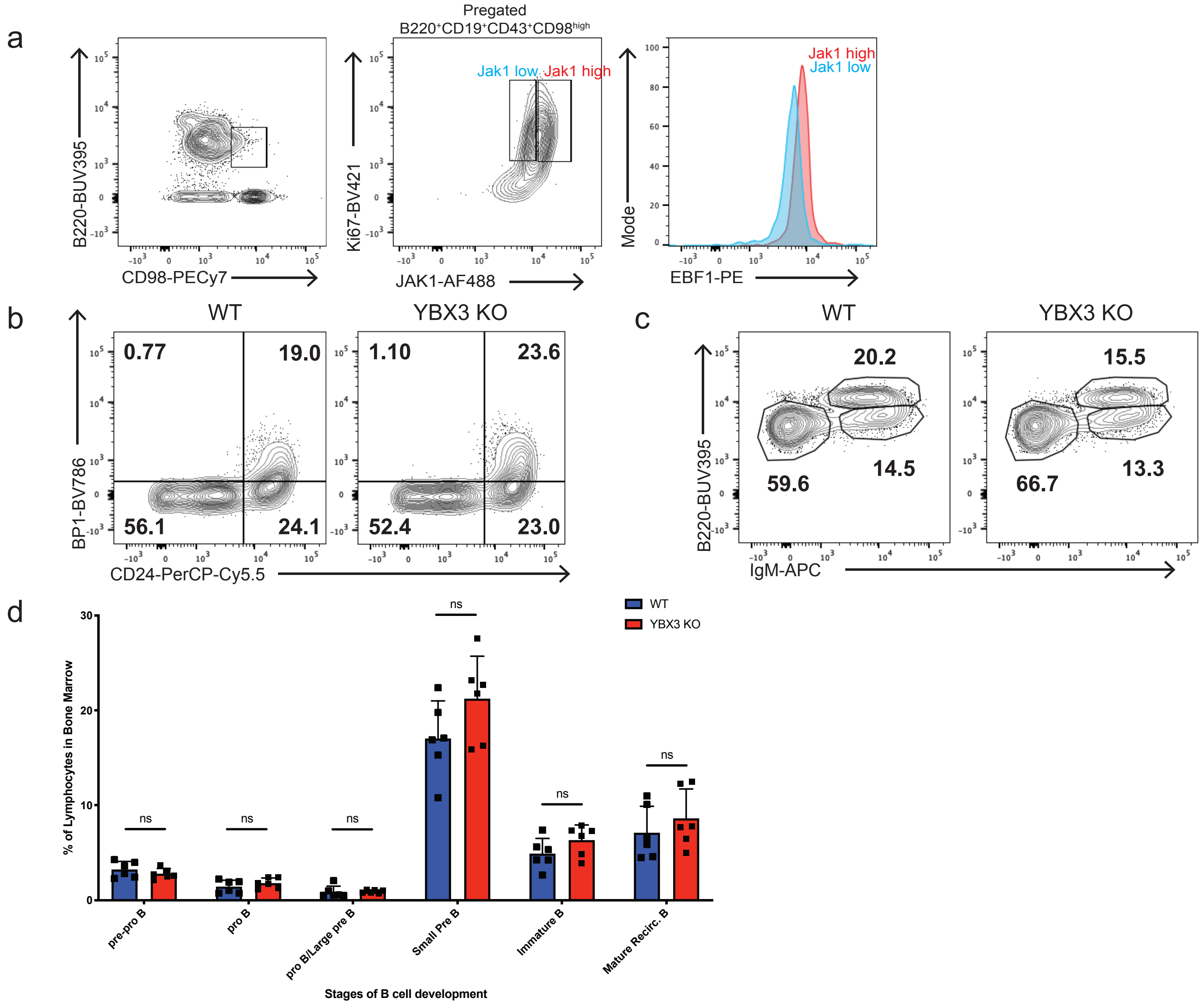

Supplementary Figure 4. Flow cytometric characterization of the role of YBX3. a. Flow cytometry analysis of WT developing B cells in the bone marrow gated on B220$^+$ and CD98^{high} cells (left). B220$^+$CD19$^+$CD43$^+$CD98^{high} cells were gated and displayed for KI67 and JAK1 expression (middle). JAK1 expression was split into low and high subsets and EBF1 expression analyzed (right). **b.** Flow cytometric analysis of B cell development from wildtype and Ybx3$^{-/-}$ mice pregated on B220$^+$CD43$^+$ progenitor B cells and stained for BP1 and CD24 expression (Hardy Fractions A-C). **c.** Flow cytometric analysis of B cell development from wildtype and Ybx3$^{-/-}$ mice pregated on B220$^+$CD43$^-$ progenitor B cells and stained for B220 and IgM expression (Hardy Fractions D-F). **d.** Summarized percentage of lymphocytes for each Hardy fraction (Fraction A-F) between wildtype and YBX3$^{-/-}$ B cells. Unpaired student t-test was performed for pre-pro B (P = 0.3001), pro B (P = 0.3286), pro B/Large pre B (P = 0.8868), Small Pre B (P = 0.1154), Immature B (P = 0.1547), and Mature Recirc. B (P = 0.3928) subsets. Data are presented as mean values ± S.D. n = 6 biologically independent mice examined from three independent experiments. WT = wild-type, YBX3 KO = Ybx3 knock-out.

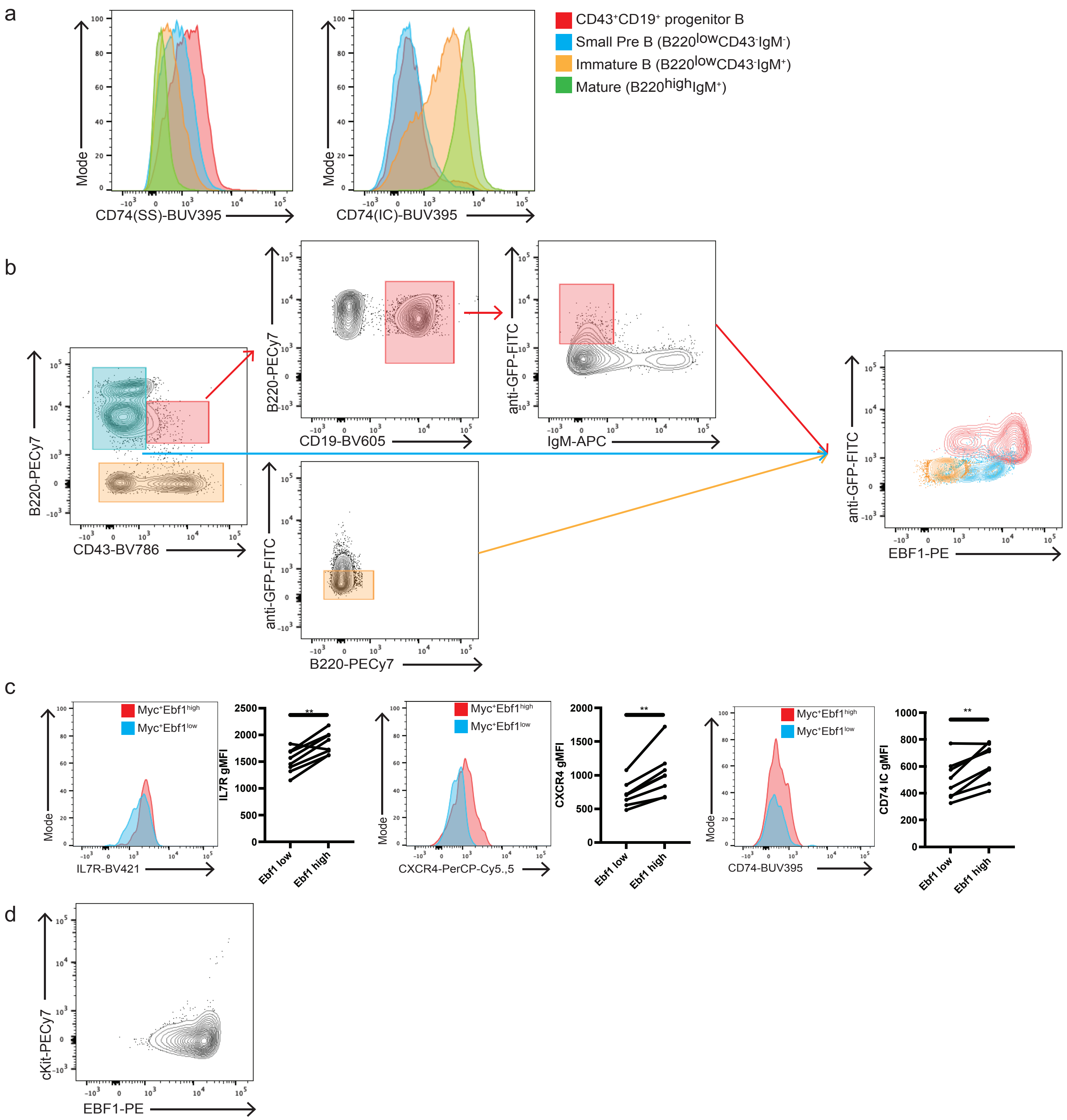

Supplementary Figure 5. Spatiotemporal regulation of CD74 expression during B cell development and EBF1 expression heterogeneity in pre-B cells. a. Representative plots of surface (left) and intracellular (right) levels of CD74 expression in developing B cell. Plots represent n = 6 mice from three independent experiments. **b.** Myc-GFP mice were used to evaluate EBF1 expression heterogeneity in preB cells. $B220^{+}CD43^{+}CD19^{+}IgM^{-}GFP^{+}$ cells were gated on (red) and evaluated for EBF1 expression. For comparison of EBF1 expression, $B220^{+}CD43^{-}$ B cells (blue) and $B220^{-}GFP^{-}$ cells (orange) were plotted together. Flow cytometry plots represent n = 8 mice from three independent experiments. **c.** Evaluation of PreBCR-dependent and PreBCR-independent cluster markers, IL7R (*P* = 0.0018), CXCR4 (*P* = 0.0011) and CD74 (*P* = 0.0025). Using the gating scheme shown in Panel B, $GFP^{+}EBF1^{low}$ (blue) and $GFP^{+}EBF1^{high}$ (red) cells were gated on and queried for IL7R, CXCR4, and CD74 expression. gMFI values are plotted from each population and connected lines represent the populations that came from the same mouse. Two-sided paired t-test was performed to determine the significance in gMFI difference between the $GFP^{+}EBF1^{low}$ and $GFP^{+}EBF1^{high}$ populations. **d.** $B220^{+}CD43^{+}CD19^{+}IgM^{-}GFP^{+}$ cells were gated on and evaluated for cKIT expression to determine the presence of pro B cells that are expressing MYC and their EBF1 expression.

a

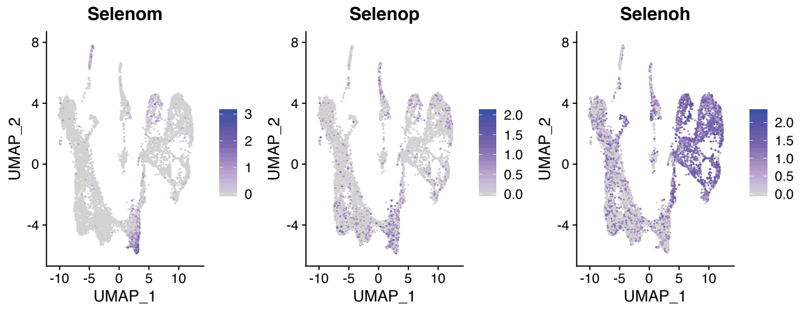

b

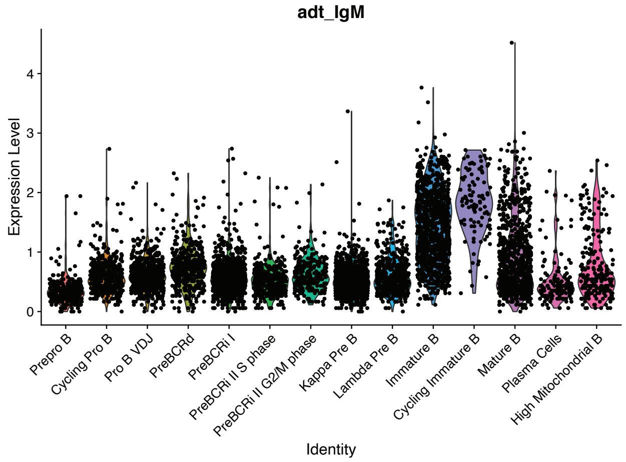

Supplementary Figure 6. Genes and surface protein expressions that are differentially expressed during B cell differentiation and maturation. a. Feature Plot of selenoprotein genes. **b.** IgM surface expression across B cell development stages. Expression level scales from a,b represent natural log transformed SCTransform corrected counts.

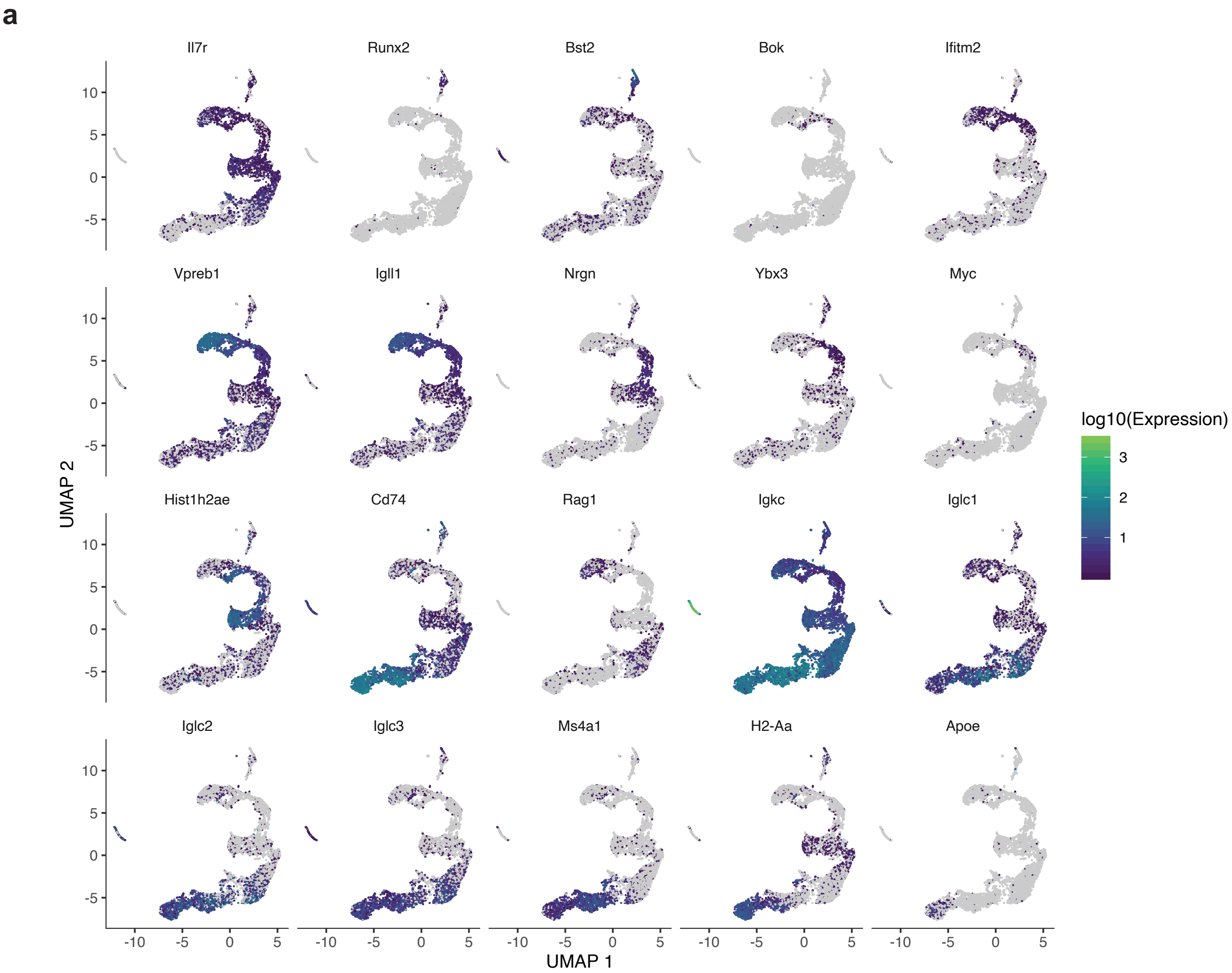

Supplementary Figure 7. Validation of cell clusters after cell-cycle regression based on stage-specific gene expression. a. Feature plots for various stage-specific genes to characterize the clusters after cell cycle gene regression

a

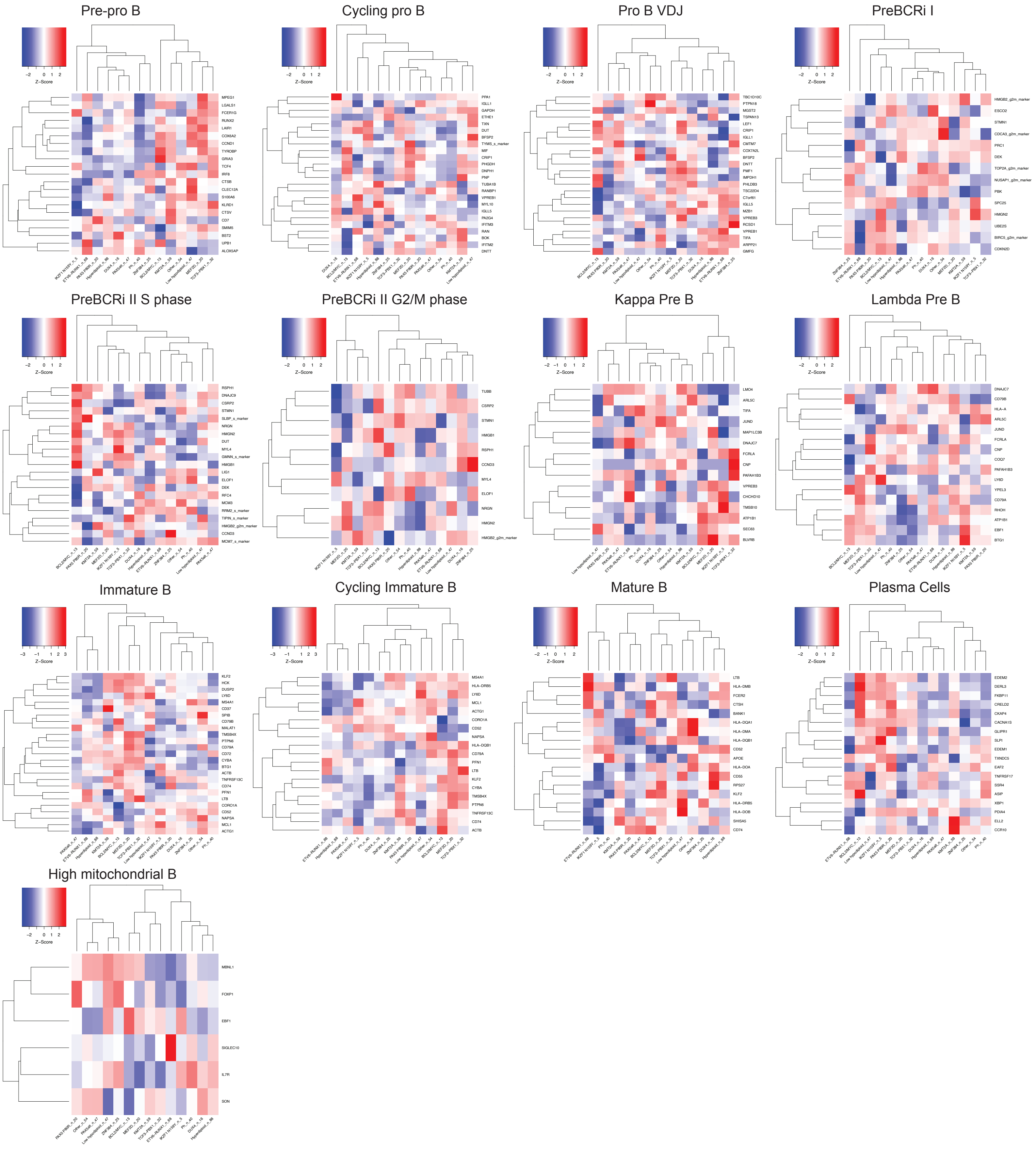

Supplementary Figure 8. Different gene expression modules from multiple stages of B cell development are exploited by various human B-ALL subtypes. a. Overlapping differentially upregulated gene markers from pre-pro B, cycling pro B, pro B VDJ, pre-BCRi I, Kappa PreB, Cycling Immature B, Mature B and Plasma cells are plotted. Gene expression intensity for each of these genes in different B-ALL subtypes is shown as a heatmap.
