# Supplementary material for: Single-cell analysis identifies dynamic gene expression networks that govern B cell development and transformation
Source: Nat Commun. 2021 Nov 25;12:6843. doi: 10.1038/s41467-021-27232-5 (PMC8617197; doi:10.1038/s41467-021-27232-5)
Supplement: Supplementary file 3 — Description of Additional Supplementary Files [file 41467_2021_27232_MOESM3_ESM.pdf]

Description of Additional Supplementary Files

File Name: Supplementary Data 1
Description: Differential gene markers of every cluster compared to all clusters. Seurat was used to identify the differential genes expressed of every cluster compared to all clusters. Avg_logFC represents log2 fold change

File Name: Supplementary Data 2
Description: Differentially expressed genes identified from pairwise comparison of each cluster. Seurat was used to identify the differential genes expressed from a pairwise comparison of each cluster. Avg_logFC represents log2 fold change

File Name: Supplementary Data 3
Description: Seurat and Monocle cluster membership and overlap. The number of cells and frequency of overlap between the Monocle and Seurat clusters are listed
