# Supplementary material for: Single-cell analysis identifies dynamic gene expression networks that govern B cell development and transformation
Source: Nat Commun. 2021 Nov 25;12:6843. doi: 10.1038/s41467-021-27232-5 (PMC8617197; doi:10.1038/s41467-021-27232-5)
Supplement: Supplementary file 7 — Reporting Summary [file 41467_2021_27232_MOESM7_ESM.pdf]

nature research

Corresponding author(s): Michael Farrar

Last updated by author(s): Oct 6, 2021

Reporting Summary

Nature Research wishes to improve the reproducibility of the work that we publish. This form provides structure for consistency and transparency in reporting. For further information on Nature Research policies, see our Editorial Policies and the Editorial Policy Checklist.

Statistics

For all statistical analyses, confirm that the following items are present in the figure legend, table legend, main text, or Methods section.

n/a	Confirmed	
☐	☒	The exact sample size (*n*) for each experimental group/condition, given as a discrete number and unit of measurement
☐	☒	A statement on whether measurements were taken from distinct samples or whether the same sample was measured repeatedly
☐	☒	The statistical test(s) used AND whether they are one- or two-sided<br>*Only common tests should be described solely by name; describe more complex techniques in the Methods section.*
☒	☐	A description of all covariates tested
☐	☒	A description of any assumptions or corrections, such as tests of normality and adjustment for multiple comparisons
☐	☒	A full description of the statistical parameters including central tendency (e.g. means) or other basic estimates (e.g. regression coefficient) AND variation (e.g. standard deviation) or associated estimates of uncertainty (e.g. confidence intervals)
☐	☒	For null hypothesis testing, the test statistic (e.g. *F*, *t*, *r*) with confidence intervals, effect sizes, degrees of freedom and *P* value noted<br>*Give P values as exact values whenever suitable.*
☒	☐	For Bayesian analysis, information on the choice of priors and Markov chain Monte Carlo settings
☒	☐	For hierarchical and complex designs, identification of the appropriate level for tests and full reporting of outcomes
☒	☐	Estimates of effect sizes (e.g. Cohen's *d*, Pearson's *r*), indicating how they were calculated

Our web collection on statistics for biologists contains articles on many of the points above.

Software and code

Policy information about availability of computer code

Data collection	Flow cytometry data was collected using Fortessa cytometers and cell sorting was performed on FACS Aria II sorters; 10X Genomics system was used to capture cells and generate libraries. Illumina NovaSeq instrument was used for sequencing.
Data analysis	Graphpad Prism 8, FlowJo (10.6.2), CellRanger (v 3.1.0), Seurat R package (v 3.0.3), Monocle3.0.2 R package, Gene set enrichment analysis (GSEA) used annotations from MSigDB (v 7.0) and completed using "enricher" from clusterProfiler R package (v 3.14.0), DoubletFinder R package (v 2.0.3).

For manuscripts utilizing custom algorithms or software that are central to the research but not yet described in published literature, software must be made available to editors and reviewers. We strongly encourage code deposition in a community repository (e.g. GitHub). See the Nature Research guidelines for submitting code & software for further information.

Data

Policy information about availability of data

All manuscripts must include a data availability statement. This statement should provide the following information, where applicable:

- Accession codes, unique identifiers, or web links for publicly available datasets
- A list of figures that have associated raw data
- A description of any restrictions on data availability

The data supporting the findings of this study are available within the paper and supplementary information file. A reporting summary is also available in the supplementary information file. Mouse genome mm10 was used as reference sequence (https://www.ncbi.nlm.nih.gov/assembly/GCF_000001635.20/). Single-cell RNA-Seq data were deposited at Gene Expression Omnibus, with the following accession code: GSE168158 (https://www.ncbi.nlm.nih.gov/geo/query/acc.cgi?acc=GSE168158). EBF1 ChIP-Seq data for figure 3e was obtained from GSM2863146 (https://www.ncbi.nlm.nih.gov/geo/query/acc.cgi?acc=GSM2863146). Bulk

RNA-seq data for wild-type and Pax5+/- x Ebf1+/- leukemia for figure 3f was obtained from GSE148680 (https://www.ncbi.nlm.nih.gov/geo/query/acc.cgi?acc=GSE148680). Source data are provided with this paper.

Field-specific reporting

Please select the one below that is the best fit for your research. If you are not sure, read the appropriate sections before making your selection.

☒ Life sciences ☐ Behavioural & social sciences ☐ Ecological, evolutionary & environmental sciences

For a reference copy of the document with all sections, see nature.com/documents/nr-reporting-summary-flat.pdf

Life sciences study design

All studies must disclose on these points even when the disclosure is negative.

Sample size	Sample size for single-cell RNAseq (n = 2) was determined based on having a biological replicate. Sample size for flow cytometry experiments had six samples from three independent experiments as this size provided sufficient power to assess differences.
Data exclusions	Excluded data includes single-cell RNA-seq data with filtered cells that did not meet minimum quality thresholds (ex. low genes expressed per cell, high mitochondrial gene percentage, and doublets due based on hasthag antibody heterotypic doublets)
Replication	Single-cell RNA-sequencing was performed once with biological replicate (two wild-type samples). All flow cytometry experiments were three independent experiments.
Randomization	Animals and samples were randomly selected for experiments.
Blinding	Investigators were not blinded to the genotype of the mice (Ybx3 WT vs KO) to ascertain phenotypic differences from gene knockout. Bioinformaticists were not blinded to sample origin during single-cell RNA-seq analysis in order to identify the genotype based on the hashtag antibodies.

Reporting for specific materials, systems and methods

We require information from authors about some types of materials, experimental systems and methods used in many studies. Here, indicate whether each material, system or method listed is relevant to your study. If you are not sure if a list item applies to your research, read the appropriate section before selecting a response.

Materials & experimental systems

n/a	Involved in the study	
☐	☒	Antibodies
☒	☐	Eukaryotic cell lines
☒	☐	Palaeontology and archaeology
☐	☒	Animals and other organisms
☒	☐	Human research participants
☒	☐	Clinical data
☒	☐	Dual use research of concern

Methods

n/a	Involved in the study	
☒	☐	ChIP-seq
☐	☒	Flow cytometry
☒	☐	MRI-based neuroimaging

Antibodies

Antibodies used

anti-mouse TotalSeq-A0301 (M1/42) Hashtag 1 antibody, BioLegend, 155801, 1 ug
anti-mouse TotalSeq-A0302 (M1/42) Hashtag 2 Antibody, BioLegend, 155803, 1 ug
anti-mouse TotalSeq-A0103 B220/CD45R (RA3-6B2), BioLegend, 103263, 1ug
anti-mouse TotalSeq-A0093 CD19 (6D5), BioLegend, 115559, 1 ug
anti-mouse TotalSeq-A0113 CD93 (AA4.1), BioLegend, 136513, 1 ug
anti-mouse TotalSeq-A0097 CD25 (PC61), BioLegend, 102055, 1 ug
anti-mouse TotalSeq-A450 IgM (RMM-1), BioLegend, 406535, 1 ug
TotalSeq-A0951 PE Streptavidin, 405251, 1 ug
anti-mouse CD19 (6D5) Brilliant Violet 605, BioLegend, 115540, 1:100
anti-mouse CD43 (S7) Biotin, BDBiosciences, 553269, 1:100
anti-mouse B220/CD45R (RA3-6B2) BUV395, BDBiosciences, 563793, 1:100
anti-mouse B220/CD45R (RA3-6B2) Pacific Blue, BDBiosciences, 558108, 1:100
anti-mouse CD11c (N418) APC-eFluor 780, ThermoFisher Scientific, 47-0114-82, 1:100
anti-mouse CD11c (N418) Brilliant Ultraviolet 395, BD Biosciences, 744180, 1:100
anti-mouse Ter119 (TER-119) APC-eFluor 780, ThermoFisher Scientific, 47-5921-82, 1:100
anti-mouse NK1.1 (PK136) APC-eFluor 780, ThermoFisher Scientific, 47-5941-82, 1:100
anti-mouse NK1.1 (PK136) PE, eBioscience, 12-5941-82, 1:100
anti-mouse Ly-6G/GR1 (RB6-8C5) APC-eFluor 780, ThermoFisher Scientific, 47-5931-82, 1:100

anti-mouse Ly-6G/GR1 (RB6-8C5) Brilliant Violet 421, BioLegend, 127627, 1:100
anti-mouse CD4 (GK1.5) APC-eFluor 780, ThermoFisher Scientific, 47-0041-82, 1:100
anti-mouse CD8a (53-6.7) APC-eFluor 780, ThermoFisher Scientific, 47-0081-82, 1:100
anti-mouse JAK1 (413104) AF488, RND Systems, IC4260G, 1:100
anti-mouse EBF1 (T26-818) PE, BDBiosciences, 565494, 1:100
anti-mouse CD127/IL7R (A7R34) BV421, BioLegend, 135023, 1:100
anti-mouse CD43 (S7) BV786, BDBiosciences, 740857, 1:100
anti-mouse CD45R/B220 (RA3-6B2) PE-Cy7, BioLegend, 103222, 1:100
anti-mouse CD184/CXCR4 (L276F12) PerCP-Cy5.5, BioLegend, 146509, 1:100
anti-mouse CD74 (In-1) BUV395, BDBiosciences, 740274, 1:100
anti-mouse CD98 (RL388) PE/Cyanine 7, BioLegend, 128124, 1:100
anti-mouse CD117/cKit (2B8) PE/Cyanine 7, eBioscience, 25-1171-82, 1:100
anti-GFP FITC, Rockland Antibodies, 600-402-215, 1:400
GhostDye Red 780, Tonbo Biosciences, 13-0865, 1:1000

Validation

All antibodies used are commercially available antibodies validated by and used per manufacturer's instructions. Antibodies from ThermoFisher, Biolegend, eBioscience, RND Systems, Rockland and Tonbo Biosciences validate antibodies for specificity with cell lines, primary cells using relevant controls across multiple immunoassays for functional application validation. All antibodies used were subject to quality control from all vendors.

Animals and other organisms

Policy information about studies involving animals; ARRIVE guidelines recommended for reporting animal research

Laboratory animals

All animals used were bred and housed in specific pathogen-free facilities at the University of Minnesota and Washington University in St. Louis and animal experiment protocols were approved by Institutional Animal Care and Use Committees (IACUC 2010-38515A and IACUC 1904-36975A). All of the animals used were 6- to 12-week old C57BL/6J males and females with appropriate age- and sex-matched controls. Specifically, the two wild-type mice used for the single-cell RNA-seq experiment were C57BL/6J males, CD45.2, and 8-week-old mice. The mice used for assessing B cells that were potentially excluded from analysis due to flow sorting gating scheme, were C57BL/6J males, CD45.2, that were 7-10 weeks old. The JAK1 and EBF1 flow experiment was performed with C57BL/6J male and female mice, CD45.1 and CD45.2, that were 7 weeks old. The Ybx3-/- phenotyping experiments were performed with C57BL/6J male and female mice, CD45.2, and 6-12 weeks old. Myc-GFP mouse experiments were performed with C57/BL6 male and female mice, CD45.2, that were 8-10 weeks old. The Ybx3-/- mice were graciously provided by Dr. Timothy Ley at Washington University in St. Louis and have been previously described36. Myc-GFP reporter mice were obtained from The Jackson Laboratory. All animals were housed in a dark/light cycle of 14 hrs/10 hrs. Light cycle was from 6AM to 8 PM. Ambient temperature was at 72Fo with a humidity ranging from 30-40%.

Wild animals

This study did not involve wild animals

Field-collected samples

This study did not involve field-collected samples

Ethics oversight

Animal experiment protocols were approved by Institutional Animal Care and Use Committees at the University of MInnesota

Note that full information on the approval of the study protocol must also be provided in the manuscript.

Flow Cytometry

Plots

Confirm that:

☒ The axis labels state the marker and fluorochrome used (e.g. CD4-FITC).

☒ The axis scales are clearly visible. Include numbers along axes only for bottom left plot of group (a 'group' is an analysis of identical markers).

☒ All plots are contour plots with outliers or pseudocolor plots.

☒ A numerical value for number of cells or percentage (with statistics) is provided.

Methodology

Sample preparation

For flow cytometry and cell sorting experiments, bilateral femurs and tibias were harvested from mice. Bones were flushed with 1X PBS with 2% fetal bovine serum (FBS), 0.1% sodium azide and 0.5 mM ethylenediaminetetraacetic acids, pH 7.4. The cells were filtered through a 70 µm mesh, centrifuged at 350 x g for 5 minutes and then incubated for 5 minutes with 5 mL of ACK lysis buffer for red blood cell lysis. Cells were then washed and centrifuged at 350 x g and subsequently resuspended for cell counting on a hemocytometer (Fisher Scientific) and staining.

Instrument

Fortessa, Aria II

Software

FlowJo (10.7.1)

Cell population abundance

Representative sorting data is present in Supplementary Figure 1. To keep the purity of B cells, we used a dump gate which included antibodies against CD11c, Ter119, NK1.1, Ly6G, CD4, CD8, and Live/dead and gated on B220+Dump- cells. B220

+Dump+ cells were 3.5%. As some B cells can express CD11c or Ly6G, we evaluated the B cells that were excluded in this analysis from this sorting scheme, which ranged from 1-3% ((Supplementary Fig. 1b)

Gating strategy	For the single-cell RNA-sequencing experiment, lymphocytes were identified by SSC-A vs FSC-A, then singlets with FSC-H, FSC-W and SSC-H and SSC-W. Singlets were then gated on B220-Pacific Blue positive cells and negative for Dump-APCef780 (CD11c, Ter119, NK1.1, Ly-6G, CD4, CD8a). Cells were then sorted on CD43+ and CD43- as shown in Figure 1.<br>For YBX3 -/- phenotyping, Hardy fractions were used to assess B cell development stages (Rumfelt et. al, 2006). In short, cells were gated on SSC-A vs FSC-A for lymphocytes then singlets were gated on SSC-A vs SSC-W. Cells were then gated on CD43+ vs CD43-. The CD43+ fraction was divided into Fraction A (CD24-BP1-),B (CD24+BP1-), and C (CD24+BP1+) using CD24 and BP1. The CD43- population was subdivided into Fraction D (B220lowIgM-), E (B220lowIgM+), and F (B220highIgM+).<br>For Myc-GFP mouse characterization, cells were gated accordingly as shown in Supplementary Figure 5. In short, cells were gated on SSCA vs FSC-A for lymphocytes then signlets were gated on SSC-A vs SSC-W. Cells were then gated on GhostRed780 (Live/dead) negative population for live cells. Cells were subsequently gated on CD43+CD19+IgM-MYCGFP+ population and evaluated for EBF1 expression, and cKIT expression

☒ Tick this box to confirm that a figure exemplifying the gating strategy is provided in the Supplementary Information.
